# *N* -butyldeoxynojirimycin reduces growth and ganglioside content of experimental mouse brain tumours

**DOI:** 10.1054/bjoc.2000.1713

**Published:** 2001-04

**Authors:** M K Ranes, M El-Abbadi, M G Manfredi, P Mukherjee, F M Platt, T N Seyfried

**Affiliations:** Department of Biology, Boston College, Chestnut Hill, MA 02467, USA; Department of Biochemistry, Glycobiology Institute, University of Oxford, South Parks Road, Oxford, OX1 3QU, UK

**Keywords:** glycosphingolipids, *N*-butyldeoxynojirimycin, glucosyltransferase, brain tumours, mouse, gangliosides, glioma

## Abstract

Abnormalities in glycosphingolipid (GSL) biosynthesis have been implicated in the oncogenesis and malignancy of brain tumours. GSLs comprise the gangliosides and the neutral GSLs and are major components of the cell surface glycocalyx. *N* -butyldeoxynojirimycin (*N* B-DNJ) is an imino sugar that inhibits the glucosyltransferase catalysing the first step in GSL biosynthesis. The influence of *N* B-DNJ was studied on the growth and ganglioside composition of two 20-methylcholanthrene-induced experimental mouse brain tumours, EPEN and CT-2A, which were grown in vitro and in vivo. *N* B-DNJ (200 μM) inhibited the proliferation of the EPEN and CT-2A cells by 50%, but did not reduce cell viability. The drug, administered in the diet (2400 mg kg^−1^) to adult syngeneic C57BL/6 mice, reduced the growth and ganglioside content of subcutaneous and intracerebral EPEN and CT-2A tumours by at least 50% compared to the untreated controls. *N* B-DNJ treatment also shifted the relative distribution of tumour gangliosides in accordance with the depletion of metabolic substrates. Side effects of *N* B-DNJ treatment were generally mild and included reductions in body and spleen weights and intestinal distension. We conclude that *N* B-DNJ may inhibit tumour growth through an effect on ganglioside biosynthesis and may be useful as a new chemotherapy for brain tumours. © 2001 Cancer Research Campaign http://www.bjcancer.com


*British Journal of Cancer* (2001) **84**(8), 1107–1114
© 2001 Cancer Research Campaign
doi: 10.1054/ bjoc.2001.1713, available online at http://www.idealibrary.com on IDEAL®

### *N*-butyldeoxynojirimycin reduces growth and ganglioside content of experimental mouse brain tumours

**MK Ranes[1], M El-Abbadi[1], MG Manfredi[1], P Mukherjee[1], FM Platt[2] and TN Seyfried[1]**

[1]Department of Biology, Boston College, Chestnut Hill, MA 02467, USA; [2]Glycobiology Institute, Department of Biochemistry, University of Oxford, South Parks Road, Oxford OX1 3QU UK

**Summary** Abnormalities in glycosphingolipid (GSL) biosynthesis have been implicated in the oncogenesis and malignancy of brain tumours. GSLs comprise the gangliosides and the neutral GSLs and are major components of the cell surface glycocalyx. *N*-butyldeoxynojirimycin (*N*B-DNJ) is an imino sugar that inhibits the glucosyltransferase catalysing the first step in GSL biosynthesis. The influence of *N*B-DNJ was studied on the growth and ganglioside composition of two 20-methylcholanthrene-induced experimental mouse brain tumours, EPEN and CT-2A, which were grown in vitro and in vivo. *N*B-DNJ (200 μM) inhibited the proliferation of the EPEN and CT-2A cells by 50%, but did not reduce cell viability. The drug, administered in the diet (2400 mg $kg^{-1}$) to adult syngeneic C57BL/6 mice, reduced the growth and ganglioside content of subcutaneous and intracerebral EPEN and CT-2A tumours by at least 50% compared to the untreated controls. *N*B-DNJ treatment also shifted the relative distribution of tumour gangliosides in accordance with the depletion of metabolic substrates. Side effects of *N*B-DNJ treatment were generally mild and included reductions in body and spleen weights and intestinal distension. We conclude that *N*B-DNJ may inhibit tumour growth through an effect on ganglioside biosynthesis and may be useful as a new chemotherapy for brain tumours. © 2001 Cancer Research Campaign http://www.bjcancer.com

**Keywords**: glycosphingolipids; *N*-butyldeoxynojirimycin; glucosyltransferase; brain tumours; mouse; gangliosides; glioma

Despite advances in surgical technique and radiation therapy, the prognosis for malignant glioma patients remains dismal, with a 2-year survival rate of less than 20% (Prados and Levin, 2000). Among the brain tumours, malignant gliomas represent 13–22% and are the leading cause of death from cancer in persons 15 to 34 years of age (Harbaugh and Black, 1998). Brain tumours are the second leading cause of cancer death in children in the United States, and their incidence is on the rise (Schmidt, 1998). Presently, the medical treatment of cancer is based on surgery and radiation therapy which may or may not be followed by chemotherapy (Shapiro, 1999; Yung et al, 2000). However, invasiveness combined with multidrug resistance protects most malignant gliomas from direct chemical, radiological or surgical assault (Harbaugh and Black, 1998). Since malignant brain tumours remain as lethal today as they were in the 1970s, new therapeutic approaches are urgently needed (Davis et al, 1999; Fathallah-Shaykh, 1999).

The glycosphingolipids (GSLs) comprise the gangliosides and the neutral GSLs and are anchored in the outer surface of plasma membranes through a lipophilic ceramide tail (Hakomori, 1996; Bai and Seyfried, 1997). Gangliosides differ from the neutral GSLs in having at least one sialic acid molecule attached to the oligosaccharide head group. N-acetylneuraminic acid (NeuAc) and N-glycolylneuraminic acid are the major sialic acid species present in mouse tissues. Chemotherapy through slowing GSL synthesis has been proposed based on evidence that GSLs may modulate tumour growth, metastasis, immunity and multidrug resistance (Chang et al, 1997; Liu et al, 1999; Radin, 1999). Defects in GSL biosynthesis are found in most human cancers and may underlie the invasive and malignant properties of brain tumours (Merzak et al, 1995; Hakomori, 1996; Berra et al, 1997). GSLs can modulate the function of growth factor receptors and have recently been implicated as modulators of brain tumour angiogenesis and cell adhesion (Hakomori, 1996; Alessandri et al, 1997; Bai and Seyfried, 1997; Manfredi et al, 1999; Zeng et al, 2000). GSLs newly synthesized and shed by tumours can competitively bind to interleukin-2 (IL-2) receptors on T cells, hindering the proliferative response required for immunological rejection (Chang et al, 1997). Taken together, these findings suggest that the modification of GSL biosynthesis and expression could be effective in brain tumour therapy.

Mammalian GSLs are synthesized by the stepwise addition of sugar residues to the oligosaccharide head group. This is accomplished through the action of a Golgi-bound multi-glycosyltransferase system, where the GSL product of one transferase serves as the substrate for another transferase (Sandhoff and van Echten, 1994; Seyfried et al, 1994). The small imino sugar, *N*B-DNJ, inhibits the ceramide-specific glucosyltransferase (GlcT-1) that catalyses the first step in GSL biosynthesis (Platt et al, 1994) (Figure 1). Since GlcCer is the common metabolic precursor for the synthesis of most gangliosides and neutral glycolipids, *N*B-DNJ treatment significantly reduces the content of all GSLs containing the GlcCer core structure (Platt et al, 1994). *N*B-DNJ is a derivative of the naturally occurring plant and microbial compound deoxynojirimycin (DNJ), and is water soluble in contrast to other, highly hydrophobic inhibitors of GSL

*Received 24 August 2000*
*Revised 10 January 2001*
*Accepted 15 January 2001*

*Correspondence to:* TN Seyfried

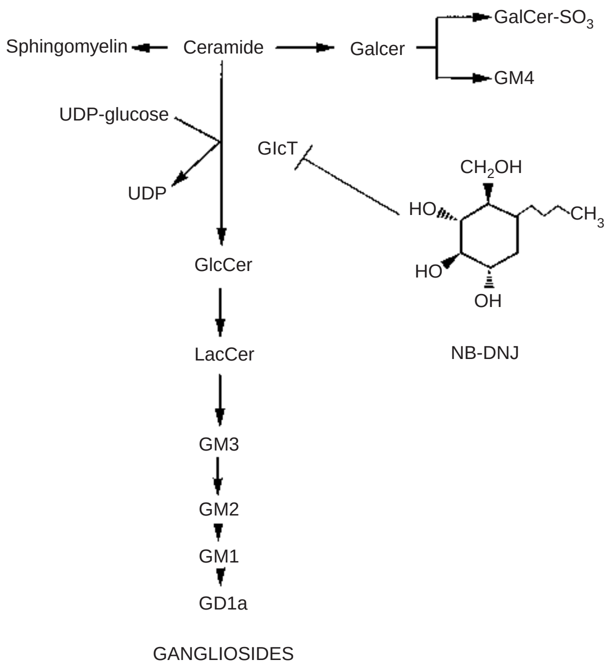

**Figure 1** Influence of *N*B-DNJ on ganglioside biosynthesis. *N*B-DNJ inhibits the ceramide-specific glucosyltransferase (GlcT) that catalyses the first committed step in GSL biosynthesis. GlcCer, glucosylceramide; LacCer, lactosylceramide; GalCer, galactosylceramide; GalCer-$SO_3$, GalCer sulfate; GlcT, glucosyltransferase biosynthesis (Platt and Butters, 1995). *N*B-DNJ is easily administered to adult mice in the diet without toxicity. Dietary administration of *N*B-DNJ prevents ganglioside GM2 accumulation in the brains of mice with Tay Sachs and Sandhoff diseases and depletes GSLs in metabolically active tissues of adult mice (Platt et al, 1994, 1997a, 1997b; Platt and Butters, 1995; Jeyakumar et al, 1999). *N*B-DNJ is therefore ideally suited as a pharmacological agent for targeting GSL biosynthesis in vivo.

*N*B-DNJ was used previously in clinical trials for Human Immunodeficiency Virus (HIV) infection and is currently used in clinical trials for the lipid storage disorder Gaucher's disease (Fischl et al, 1994; Cox et al, 2000). In this study, we have investigated for the first time the influence of *N*B-DNJ on the growth and ganglioside content of two 20-methylcholanthrene (20 MC)-induced mouse brain tumours, EPEN and CT-2A. The 20-MC-induced murine brain tumours share a number of morphological, physiological, and histological features with malignant brain tumours in humans (Zimmerman, 1982; Seyfried et al, 1992; Seyfried, 2001). Our present results demonstrate that *N*B-DNJ inhibits the growth and ganglioside content of both the EPEN and the CT-2A experimental brain tumours. These findings suggest that *N*B-DNJ may have clinical efficacy in treating malignant brain cancer in humans. A preliminary report of these findings has appeared (Ranes et al, 2000).

### MATERIALS AND METHODS

### Mice

The C57BL/6 (B6) strain was obtained from the Jackson Laboratory (Bar Harbor, ME). All B6 mice used in this study were propagated in the animal room of the Biology Department, Boston College, using animal husbandry conditions described previously (Flavin et al, 1991). Age- and sex-matched (6–8 week-old male) B6 mice were used as tumour recipients. All animal use procedures were in accordance with the NIH Guide for the Care and Use of Laboratory Animals and were approved by the Institutional Animal Care Committee.

### Experimental mouse brain tumours

Both EPEN and CT-2A experimental mouse brain tumours were used for these studies. The EPEN tumour was originally produced through implantation of 20-MC into the cerebral ventricle of a B6 mouse and had histological characteristics of an ependymoblastoma (Rubin, 1968). The CT-2A tumour was generated in our laboratory and arose in a B6 mouse 450 days after 20-MC implantation into the cerebral cortex as previously described (Seyfried et al, 1992). Histologically, the CT-2A brain tumour was broadly classified as a poorly differentiated highly malignant anaplastic astrocytoma. The EPEN tumour grows as a firm, cohesive, nonhaemorrhagic mass in vivo and as cell islands in vitro, while the CT-2A tumour grows as a soft, noncohesive, haemorrhagic mass in vivo and as a fusiform monolayer in vitro (Seyfried et al, 1992). Also, the growth rate is significantly greater for the CT-2A tumour than for the EPEN tumour (Cotterchio and Seyfried, 1993).

### *N*-butyldeoxynojirimycin

*N*B-DNJ (OGT918; 219.3, MW) was obtained as a gift from Searle/Monsanto, St Louis, MO, and Oxford GlycoSciences, Abigdon, UK.

### Cell culture and *N*B-DNJ treatment

Approximately $2 \times 10^3$ EPEN or CT-2A tumour cells were seeded in wells of a 24-well plate and cultured for 24 h in a humidified atmosphere of 95% air and 5% $CO_2$ at 37˚C in Dulbecco's modified Eagle medium (DMEM) supplemented with 10% heat-inactivated fetal bovine serum (FBS). Cells were cultured for an additional 8 days in the presence or absence of 200 μM *N*B-DNJ. A Coulter counter was used for cell counts of the control and treated cells, and the trypan blue exclusion procedure was used to assess the viability of tumour cells following *N*B-DNJ treatment (Kemp, 1968). The number of dead cells that incorporated trypan blue was counted using a haemacytometer.

### Subcutaneous and intracerebral tumour implantation

An EPEN or CT-2A flank-grown tumour was removed from a B6 mouse and was rinsed and diced in cold phosphate-buffered saline (PBS) at pH 7.4. The right flanks of recipient mice were shaved in order to facilitate tumour inoculation, detection, and growth assessment. Mice were anaesthetized with metofane (Methoxyflurane, Mallinckrodt Veterinary Inc, Mundelein, IL), and the tumour was implanted by the s.c. injection of 0.1 ml of minced tumour tissue suspended in 0.2 ml PBS by use of a 1 cc tuberculin syringe and 18-gauge needle. The inoculated mice were monitored daily for nodule formation. Tumours were implanted i.c. as previously described (Seyfried et al, 1987). Mice were anaesthetized with metofane, and their heads were shaved and then swabbed with 70% ethyl alcohol under sterile conditions. A midline incision was made on the posterior aspect of the head and the periosteum was swept laterally to expose the sagittal, coronal and lamboidal sutures of the skull. A 1 mm burr hole was made, using an electric

 © 2001 Cancer Research Campaign drill, over the right parietal region behind the coronal suture and lateral to the sagittal suture. A 1 $mm^3$ tumour fragment was then implanted into the right cerebral hemisphere of each anaesthetized recipient B6 mouse using a trocar. The skin edges were immediately approximated using sterile surgical forceps and fused using collodion (JT Baker, Inc, Phillipsburg, NJ). The animals recovered spontaneously and were monitored and returned to their cages when fully active.

### In vivo *N*B-DNJ treatment and tumour growth assessment

B6 mice bearing s.c. or i.c. tumors were randomized into either control or treated groups and were housed 2 per cage. *N*B-DNJ was administered in the diet as previously described (Platt et al, 1997a, 1997b). Mice in the control group received ground mouse chow (Agway Prolab Rat/Mouse/Hamster 3000, PMI Feeds, Inc, St Louis, MO). Mice in the treated group received ground mouse chow containing *N*B-DNJ (2400 mg *N*B-DNJ kg body weight$^{-1}$ day$^{-1}$). Previous studies showed that this dietary dosage of *N*B-DNJ reduces GSL content in liver and lymphoid organs by 50–70% and is well tolerated in B6 mice over a 2-week treatment period (Platt et al, 1997b). The diet and compound (both as dry solids) were mixed thoroughly before use, stored in an airtight container at room temperature, and were used within 7 days of mixing. Both control and *N*B-DNJ-containing diets were provided ad libitum and were administered in glass vials that were fixed to the cage bottom. Water was also provided ad libitum.

Tumours grown s.c. were measured with calipers by the method of Looney as previously described (Cotterchio and Seyfried, 1993). Tumour volume was calculated by the formula for the approximation of the volume of a spheroid as Volume = 1/2 (Length × Width × Height). Tumour volume was measured every other day throughout the treatment period. Since EPEN tumour growth rate is slower than that of the CT-2A tumour, it took longer for the EPEN tumour to reach a measurable size (30–40 $mm^3$). Consequently, *N*B-DNJ treatment of the EPEN or CT-2A tumours grown s.c. was initiated on day 14 or on day 6 post-inoculation, respectively. Treatment of the EPEN or CT-2A tumours was terminated on day 28 or on day 14 post-inoculation, respectively, when the control s.c. tumours reached an approximate mean volume of 1000 $mm^3$.

*N*B-DNJ treatment was initiated 6 days after i.c. implantation for both the EPEN and the CT-2A tumours. This ensured vigorous tumour growth at the initiation of drug treatment. Unlike s.c. tumour growth, cranium size constrains i.c. tumour growth. Consequently, *N*B-DNJ treatment was terminated on day 15 post-implantation for both tumours. In addition to tumour volume measurements, mouse body weights were measured to the nearest 0.1 g and behavioural activity (ambulation and grooming) was monitored. At the termination of drug treatment, all mice were sacrificed by cervical dislocation and tumour, brain, liver and spleen tissue was collected, frozen and then lyophilized. In an independent study, healthy mice were treated with *N*B-DNJ (2400 mg kg$^{-1}$ d$^{-1}$) for 9 days to measure serum and tissue *N*B-DNJ content.

### Analysis of gangliosides

Gangliosides were isolated and purified from lyophilized tumour, brain and liver tissue by our previously described methods (Seyfried et al, 1978; Seyfried et al, 1987; El-Abbadi and Seyfried, 1994). Briefly, total lipids were extracted in chloroform:methanol (1:1, v/v). The Folch procedure was used to partition gangliosides into the upper aqueous phase (Folch et al 1957). The upper phase was converted to a chloroform:methanol:water ratio of 30:60:8 (v/v/v) for ion exchange chromatography (Seyfried et al, 1978). Samples were then applied to a DEAE-Sephadex A-25 (Pharmacia Biotech) column (acetate form) and non-acidic glycolipids were eluted by washing with chloroform:methanol: water (30:60:8, v/v/v). The gangliosides were then eluted from the column with chloroform:methanol:0.8 M sodium acetate (30:60:8, v/v/v). Residual phosphatides and ganglioside inner esters were removed by a mild base treatment in 0.1 N sodium hydroxide at 37˚ C for 1.5 h. C18 Bond Elut columns (Varian) were used to remove salts and base from ganglioside fractions. Quantification of N-acetylneuraminic acid (NeuAc) and N-glycolylneuraminic acid (NeuGc) was determined using a gas-liquid chromatography procedure (Yu and Ledeen, 1970). The resorcinol reaction was also used to analyse ganglioside sialic acid concentration in mouse brains as previously described (Seyfried et al, 1978). Sialic acid concentration was expressed as μg 100 mg dry weight$^{-1}$. The distribution of tumour gangliosides was examined using high performance thin-layer chromatography (HPTLC) as we previously described (Cotterchio and Seyfried, 1993). Ganglioside sialic acid was visualized on the HPTLC by the resorcinol-hydrochloric acid spray (Svennerholm, 1957).

### Detection of *N*B-DNJ in serum and tissue of healthy mice

Serum and tissue *N*B-DNJ levels were quantified as previously described (Andersson et al, 2000). Healthy 6–8 week-old-male B6 mice were treated for 9 days with *N*B-DNJ at 2400 mg kg$^{-1}$ d$^{-1}$. Mice were sacrificed by carbon dioxide inhalation. Serum was collected and stored at –20˚C. Brain and liver tissue was collected and stored at –80˚C. Serum and supernatant of liver homogenate (130 mg ml$^{-1}$ in 10% methanol) were centrifuged 3 times through a Millipore Ultrafree filter, after an internal standard (*N*-3-methyl-butyl-DNJ) had been added to the samples. The pooled filtrates were purified on a hydrochloric acid-preconditioned SCX column, eluted with 1% ammonia in methanol, dried down, and resuspended in water. The samples were further purified on a C18 column (methanol preconditioning, water wash, and methanol elution), and then quantified by HPLC (Dionex CS10 hpcec chromatography, isocratic elution with 50 mM sodium sulphate and 5% acetonitrile, pulsed amperometric detector).

### Histological examination

Control and *N*B-DNJ-treated brains bearing the EPEN or the CT-2A tumours were dissected and fixed in formalin for 24 hours. Specimens were embedded in paraffin and sectioned at 5 μm thickness. For routine histological examination of tumour morphology, the EPEN and the CT-2A tumour sections were stained with haematoxylin-eosin (H&E) and examined by light microscopy (Zeiss, West Germany).

### RESULTS

### Influence of *N*B-DNJ on in vitro EPEN and CT-2A cell growth

*N*B-DNJ (200 μM) significantly reduced the number of EPEN

© 2001 Cancer Research Campaign 

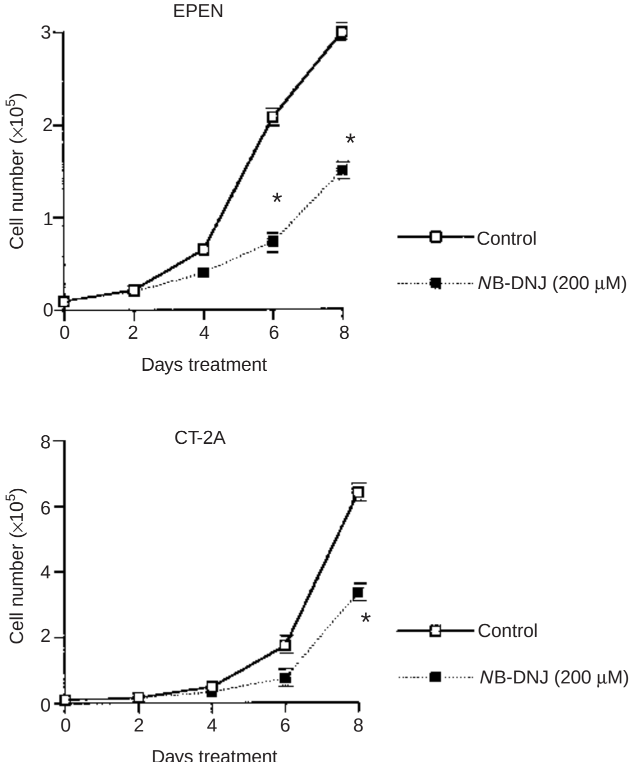

**Figure 2** Influence of *N*B-DNJ on the in vitro growth rate of EPEN and CT-2A experimental mouse brain tumour cells. Values are expressed as the means ± SEM of 3 independent experiments for each cell line. The asterisks indicate that cell counts were significantly lower in the *N*B-DNJ-treated flasks than in the control flasks at the $P < 0.01$ level (two-tailed *t*-test)

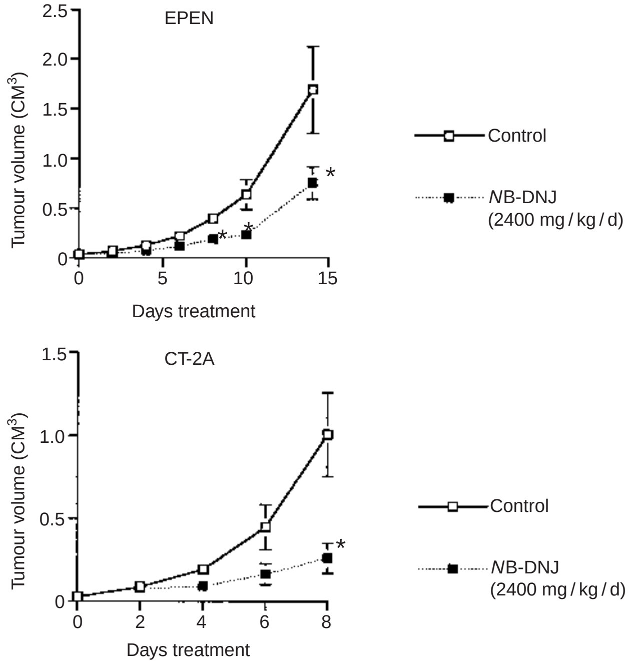

**Figure 3** Influence of *N*B-DNJ on the subcutaneous growth rate of EPEN and CT-2A experimental mouse brain tumours. Day 0 represents the beginning of tumour volume measurement, when tumours were approximately 30–40 $mm^3$. The values for each tumour (EPEN control, $n = 4$; EPEN + *N*B-DNJ, $n = 4$; CT-2A control, $n = 7$; CT-2A + *N*B-DNJ, $n = 7$) are expressed as means ± SEM. The asterisks indicate that *N*B-DNJ-treated tumour volumes were significantly lower than control tumour volumes at the $P < 0.01$ level (two-tailed *t*-test)

cells by 50% and the number of CT-2A cells by 48% compared to untreated cells after 8 days of treatment (Figure 2). *N*B-DNJ was not cytotoxic since the percentage of trypan blue positive cells (2–3%) was similar in both the control and treated flasks for both tumour lines. These data indicate that *N*B-DNJ inhibited EPEN and CT-2A cell proliferation without enhancing cytotoxicity.

### Influence of *N*B-DNJ on the EPEN and the CT-2A tumour growth in vivo

Based on the anti-proliferative effect of *N*B-DNJ on EPEN and CT-2A tumour cells in vitro, we examined the effect of *N*B-DNJ on the growth of both brain tumours in vivo. *N*B-DNJ (2400 mg $kg^{-1}$ $day^{-1}$) significantly reduced both s.c. and i.c. growth of the EPEN and the CT-2A tumours (Figures 3 and 4). The inhibitory effect of *N*B-DNJ treatment on s.c. growth was apparent for both tumours by the fourth or sixth day of treatment. By the end of treatment, the EPEN and the CT-2A tumour volumes were reduced by 55% and 72%, respectively, compared to tumours in the untreated control mice (Figure 3). The greater inhibitory effect of *N*B-DNJ on the CT-2A tumours than on the EPEN tumours may result from the faster growth rate of the CT-2A tumours compared to the EPEN tumours.

Total tumour dry weight was used to determine i.c. growth at the end of the treatment period. Dry weight provides a more accurate assessment of tumour growth than wet weight because it eliminates variability due to differences in tissue water content associated with oedema. The dry weights of the *N*B-DNJ-treated EPEN and CT-2A tumours were reduced by 71% and 55%, respectively, compared to tumours in the untreated control mice (Figure 4). The

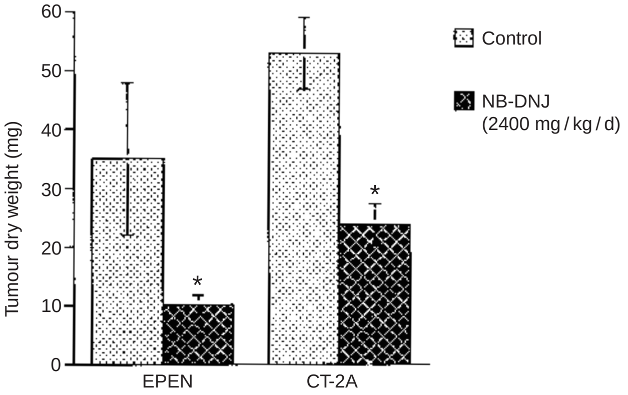

**Figure 4** Influence of *N*B-DNJ on the intracerebral growth of EPEN and CT-2A experimental mouse brain tumours. Tumour dry weights (EPEN control, $n = 4$; EPEN + *N*B-DNJ, $n = 7$; CT-2A control, $n = 8$; CT-2A + *N*B-DNJ, $n = 8$) are expressed as the means ± SEM. The asterisks indicate that *N*B-DNJ-treated tumour dry weights were significantly lower than control tumour dry weights at the $P < 0.01$ level (two-tailed *t*-test)

greater inhibitory effect of *N*B-DNJ on the EPEN tumours than on the CT-2A tumours may result from the smaller size of the EPEN than the CT-2A tumour at the initiation of drug treatment (day 6). Taken together, these findings indicate that *N*B-DNJ effectively inhibits the growth of the EPEN and the CT-2A tumours in both subcutaneous and intracerebral environments.

### Influence of *N*B-DNJ on tumour, liver and brain ganglioside content

Since *N*B-DNJ is a known inhibitor of GSL biosynthesis, we

 © 2001 Cancer Research Campaign

**Table 1** Influence of NB-DNJ on ganglioside content in experimental mouse brain tumours and liver

| Tissue Growth Environment | Treatment | $N^a$ | Total | Ganglioside neuraminic acid content, μg 100 mg$^{-1}$ dry weight | | |
|---|---|---|---|---|---|---|
| | | | | NeuAc[b] | NeuGc | % NeuGc |
| EPEN | | | | | | |
| SC[c] | Control | 4 | 44.4 ± 5.2 | 38.1 ± 5.1 | 6.3 ± 1.1 | 14 |
| | NB-DNJ | 6 | 19.5 ± 2.0** | 16.0 ± 1.6** | 3.5 ± 0.5* | 18 |
| IC | Control | 3 | 68.5 ± 0.6 | 60.5 ± 1.2 | 8.0 ± 0.9 | 12 |
| | NB-DNJ | 3 | 35.0 ± 3.5** | 32.6 ± 2.9** | 2.4 ± 0.5* | 7 |
| CT-2A | | | | | | |
| SC | Control | 6 | 62.2 ± 5.9 | 39.2 ± 3.8 | 23.1 ± 4.0 | 37 |
| | NB-DNJ | 6 | 37.8 ± 4.0* | 28.7 ± 2.7 | 9.0 ± 1.3* | 24 |
| IC | Control | 6 | 61.6 ± 3.3 | 43.1 ± 3.7 | 18.5 ± 1.3 | 30 |
| | NB-DNJ | 6 | 30.9 ± 2.9** | 25.2 ± 2.7* | 5.7 ± 0.9** | 18 |
| Liver | | | | | | |
| | Control | 3 | 30.5 ± 2.9 | 1.95 ± 0.2 | 28.6 ± 2.7 | 94 |
| | NB-DNJ | 4 | 11.9 ± 1.2** | 2.7 ± 0.2 | 9.2 ± 1.2** | 77 |

[a]*N*, number of independent tumour or liver samples studied. Values are expressed as means ± the SEM. [b]NeuAc, *N*-acetylneuraminic acid; NeuGc, *N*-glycolylneuraminic acid. [c]IC, intracerebral; SC, subcutaneous. Asterisks indicate that the control and treated tissue differ at the $P < 0.01$ (*) and $P < 0.001$ (**) levels as determined by the two-tailed *t* test.

compared tumour, liver and brain ganglioside content in untreated and *N*B-DNJ-treated mice. *N*B-DNJ significantly reduced the total ganglioside content of both the EPEN and CT-2A tumours grown either s.c. or i.c. (Table 1). Ganglioside concentration in the *N*B-DNJ-treated EPEN and CT-2A tumours grown s.c. was reduced by 56% and 39%, respectively. Ganglioside concentration of the treated tumours grown i.c. was reduced by 49% and 50%, respectively. The reduction in ganglioside content was observed for gangliosides containing both NeuAc and NeuGc sialic acids. In general, *N*B-DNJ treatment caused a greater reduction in NeuGc-containing gangliosides relative to NeuAc-containing gangliosides (Table 1). *N*B-DNJ also significantly reduced total liver ganglioside content by 74% (Table 1). Although total brain ganglioside concentration (μg sialic acid 100 mg dry weight$^{-1}$ ± SEM) was about 11% lower in the NB-DNJ-treated mice (454 ± 19, $n = 3$) than in the untreated control mice (508 ± 10, $n = 3$), the difference was not statistically significant (determined by the two-tailed *t*-test). GSLs within the brain have a relatively slow turnover, rendering brain ganglioside biosynthesis less susceptible to inhibition by *N*B-DNJ. These findings suggest that *N*B-DNJ inhibition of ganglioside synthesis is greater in the more metabolically active tissues than in the less metabolically active tissues.

### Influence of *N*B-DNJ on tumour, liver and brain ganglioside distribution

The HPTLC distribution of gangliosides in the untreated EPEN and CT-2A tumours grown s.c. or i.c. was similar to the distribution that we reported previously for these tumours (Seyfried et al, 1987; Cotterchio and Seyfried, 1993; El-Abbadi and Seyfried, 1994). GM3 is the only ganglioside synthesized by cultured EPEN cells, while gangliosides in the 'a' metabolic pathway (GM3, GM2, GM1, GD1a) are the major gangliosides synthesized by cultured CT-2A cells. Most other gangliosides in the tumours are derived from tumour-infiltrating host cells or from the serum (Seyfried et al, 1996; Ecsedy et al, 1998; Seyfried et al, 1998). The significant reduction in total ganglioside content in the *N*B-DNJ-treated tumours (Table 1) was associated with a noticeable shift in ganglioside distribution from the structurally simple (GM3) to the structurally complex gangliosides (GD1a, GT1b) (Figure 5). *N*B-DNJ treatment had no significant effect on the distribution of brain or liver gangliosides (data not shown).

### *N*B-DNJ content in serum and tissue of healthy mice

To determine the tissue bioavailability, *N*B-DNJ levels were measured in the brain, liver and serum of healthy B6 mice after 9 days of treatment at 2400 mg kg$^{-1}$ d$^{-1}$. The concentration of *N*B-DNJ in the serum was 58 μM ± 4.4 ($n = 3$), an amount consistent with steady-state serum levels previously reported (Platt et al, 1997b). *N*B-DNJ concentration in the liver was 4.6 ± 0.7 μg g$^{-1}$ wet weight ($n = 3$), which was more than 10-fold higher than that in the brain (0.3 ± 0.1 μg g$^{-1}$ wet weight, $n = 3$). These findings suggest that *N*B-DNJ uptake is greater in the more metabolically active liver tissue than in the less metabolically active brain tissue or that the blood-brain barrier restricts brain uptake.

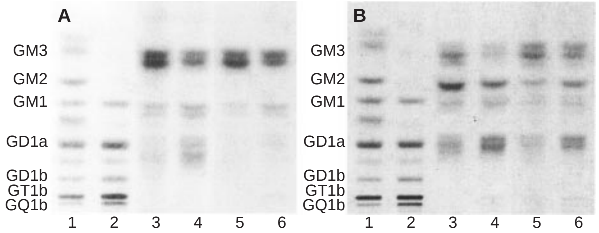

**Figure 5** High-performance thin-layer chromatogram showing the effects of *N*B-DNJ treatment on EPEN (**A**) and CT-2A (**B**) tumour ganglioside distribution. Approximately 2 μg of ganglioside sialic acid were spotted for each sample. The plate was developed by one ascending elution with chloroform:methanol:water (50:45:10, v/v/v) that contained 0.02% $CaCl_2 2H_2O$. The bands were visualized by resorcinol spray (50). In (**A**) and (**B**), lanes 1, ganglioside standards; lanes 2, adult mouse brain gangliosides; lanes 3, control s.c. tumours; lanes 4, *N*B-DNJ-treated s.c. tumours; lanes 5, control i.c. tumours; lanes 6, *N*B-DNJ-treated i.c. tumours

© 2001 Cancer Research Campaign

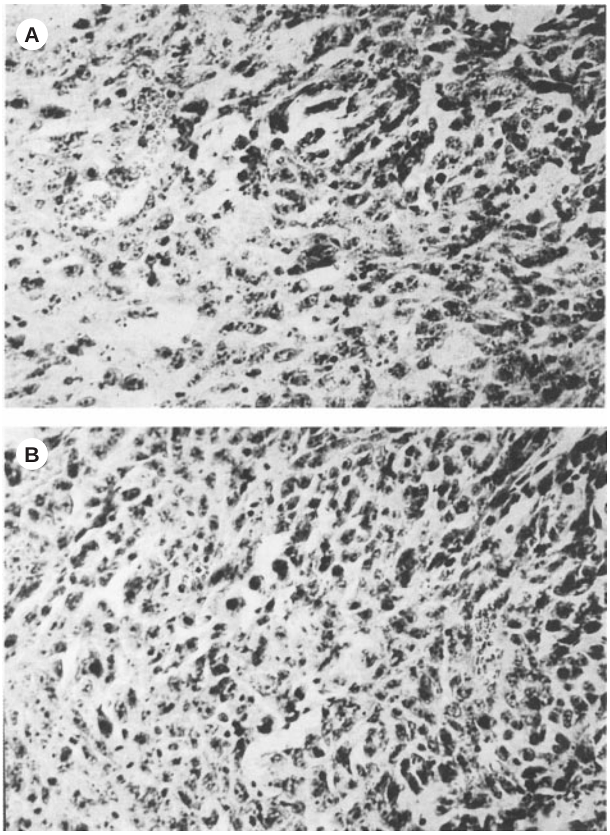

**Figure 6** Histological appearance of the CT-2A tumour grown intracerebrally in control (**A**) and in *N*B-DNJ-treated (**B**) animals. Magnification × 200

### Influence of *N*B-DNJ on EPEN and CT-2A on histological features

To ascertain whether a reduction in growth and ganglioside content may affect tissue morphology, we examined *N*B-DNJ-treated EPEN and CT-2A tumours using H&E staining. *N*B-DNJ treatment had no significant effect on the histological morphology of the EPEN tumour, which appeared as previously described (Seyfried et al, 1987). In parallel, *N*B-DNJ treatment had no significant effect on the morphology of the CT-2A tumour (Figure 6). The amount of necrosis or pyknosis was not noticeably different between the control and *N*B-DNJ-treated EPEN or CT-2A tumours.

### Side effects of *N*B-DNJ treatment

*N*B-DNJ was not overtly toxic as none of the treated mice died during the experiment. After the third day of treatment, mice receiving *N*B-DNJ had a hunched stature and dull coat in comparison to untreated control mice. The final mouse body weights were 13% lower on average in the *N*B-DNJ-treated groups, compared to the control groups. The weight loss may be associated with digestive problems since, upon dissection, the large intestines of treated mice appeared distended by the presence of gas pockets. *N*B-DNJ treatment did not affect brain dry weight, but reduced spleen dry weight by 61%, as previously reported (Platt et al, 1997b; Andersson et al, 2000). Overall, the animals tolerated *N*B-DNJ in their diet and were not seriously compromised by its administration over the short treatment period.

### DISCUSSION

Our data show that the small imino sugar, *N*B-DNJ, significantly inhibited the growth rate and the ganglioside content of 2 different experimental mouse brain tumours, the EPEN and the CT-2A. The reduction of ganglioside content in the i.c.-grown tumours and the detection of *N*B-DNJ in the brain tissue of normal mice suggests that *N*B-DNJ passes the blood–brain barrier, consistent with previous studies (Platt et al, 1997a; Jeyakumar et al, 1999; Andersson et al, 2000). In contrast to most known chemotherapeutic molecules, *N*B-DNJ was non-cytotoxic and was generally well tolerated by the B6 mouse host. Since gangliosides modulate the activities of multiple cell surface receptors and adhesion molecules that participate in cell proliferation, migration, and angiogenesis (Hakomori, 1996; Alessandri et al, 1997), it is possible that the *N*B-DNJ-linked inhibition of ganglioside biosynthesis is responsible for the reduced tumour growth. We do not exclude the possibility that *N*B-DNJ may also reduce tumour growth through effects on *N*-linked glycosylation or on caloric intake (Weindruch and Walford, 1988; Karlsson et al, 1993; Platt and Butters, 1995). It is unlikely that *N*B-DNJ reduces brain tumour growth through an effect on apoptosis because no noticeable differences were seen between the control and the *N*B-DNJ-treated tumours for the number of dead cells in vitro or for cell pyknosis or tissue necrosis in vivo. Additional studies will be needed to determine the mechanisms by which *N*B-DNJ inhibits brain tumour growth and whether reduced tumour growth and ganglioside content are correlated with *N*B-DNJ dosage.

We found that *N*B-DNJ reduced the content of NeuGc-containing gangliosides to a greater extent than that of NeuAc-containing gangliosides in the i.c.-grown brain tumours. We previously showed that most NeuGc-containing gangliosides present in mouse brain tumours and in human glioma xenografts are derived from tumour-associated host cells (macrophages) and from the mouse serum (Ecsedy et al, 1998, 1999; Seyfried et al, 1998). Since most NeuGc-containing gangliosides in mouse serum are synthesized in the liver (Cotterchio and Seyfried, 1994), we suggest that the marked reduction of NeuG-containing gangliosides in the i.c.-grown brain tumours results in large part from the *N*B-DNJ-induced inhibition of ganglioside biosynthesis in the liver. It is not yet known if *N*B-DNJ might also reduce tumour NeuGc content through an effect on tumour-associated macrophages.

We recently showed that gene-linked changes in ganglioside distribution could influence angiogenesis in the EPEN tumour (Manfredi et al, 1999). Specifically, the neosynthesis of complex gangliosides (GM2, GM1, and GD1a) with a concurrent decrease in GM3 enhanced angiogenesis. These shifts in ganglioside distribution, however, were not associated with changes in total cellular ganglioside content. Although we found similar ganglioside shifts in the *N*B-DNJ-treated tumours, these shifts were associated with blocked ganglioside synthesis and significant reductions in total ganglioside content. Further studies are needed to determine if the effects of *N*B-DNJ on tumour ganglioside distribution also influence angiogenesis.

The *N*B-DNJ-induced shift in tumour ganglioside distribution from the more simple structures (GM3) to the more complex gangliosides (GM1, GD1a) in the CT-2A tumour results from a depletion of simple upstream metabolic precursors (glucosyl-

© 2001 Cancer Research Campaign ceramide and lactosylceramide) together with the conversion of already synthesized precursors to ganglioside end products (GD1a). A similar phenomenon was described previously in mouse embryos treated with N-butyldeoxygalactonojirimycin (NB-DGJ), a structural analogue of *N*B-DNJ, and is consistent with the substrate availability hypothesis of ganglioside biosynthesis (Seyfried et al, 1994; Brigande et al, 1998). The shift in ganglioside distribution in the EPEN tumour likely results from the depletion of GM3 synthesis in the proliferating tumour cells. Such a depletion will differentially magnify those gangliosides (GM1, GD1a) in the less proliferative host cells e.g., macrophages. Hence, *N*B-DNJ may influence tumour ganglioside composition through effects on both the tumour cells and the host cells.

Mammalian systems lack enzymes that metabolize the imino sugars and, as a consequence, *N*B-DNJ is secreted intact via the kidney (Platt and Butters, 1995, 1998). For this reason, secondary metabolites do not complicate the biological effects of *N*B-DNJ. The 58 μM steady-state serum level of *N*B-DNJ that we detected in the B6 mice after 9 days of dietary administration (2400 mg $kg^{-1}d^{-1}$) was similar to that found previously for inhibition of GSL biosynthesis in normal and pathological mouse tissues (Platt et al, 1997a, 1997b; Jeyakumar et al, 1999). In these previous studies, *N*B-DNJ treatment caused extensive depletion of GSLs in the liver and the lymphoid organs of healthy B6 mice and reduced the concentration of ganglioside GM2 in the brains of Tay-Sachs and Sandhoff disease. In phase I and phase II clinical trials, *N*B-DNJ effectively reduced glucocerebroside storage in patients with Gaucher's disease, but did not slow the progression of HIV infection (Fischl et al, 1994; Cox et al, 2000). Osmotic diarrhoea was the major side effect reported in patients treated with *N*B-DNJ, and was apparently caused by the competitive inhibition of the intestinal disaccharidases, sucrase and isomaltase (Fischl et al, 1994; Platt et al, 1997b; Anderson et al, 2000). It is possible that the intestinal gas pockets in our treated mice also relate to the *N*B-DNJ-induced inhibition of digestive enzymes. Modification of *N*B-DNJ dosage and carbohydrate intake may reduce GI tract distress. In summary, our preclinical findings demonstrate for the first time that *N*B-DNJ may be an effective, orally administered chemotherapy for human brain tumours.

### ACKNOWLEDGEMENTS

We would like to thank Dr Raymond Dwek for his support, Drs Clare O'Connor and Daniel Kirschner for critical comments. We thank Oxford GlycoSciences and Searle/Monsanto for *N*B-DNJ. This research was supported by the Boston College Research Expense Fund and the University of Oxford Glycobiology Institute. FNP is a Lister Institute Research Fellow.

### REFERENCES

Alessandri G, Cornaglia Ferraris P and Gullino PM (1997) Angiogenic and angiostatic microenvironment in tumors – role of gangliosides. *Acta Oncol* **36**: 383–387

Andersson U, Butters TD, Dwek RA and Platt FM (2000) N-butyldeoxygalactonojirimycin: a more selective inhibitor of glycosphingolipid biosynthesis than N-butyldeoxynojirimycin, in vitro and in vivo. *Biochem Pharmacol* **59**: 821–829

Bai H and Seyfried TN (1997) Influence of ganglioside GM3 and high density lipoprotein (HDL) on the cohesion of mouse brain tumor cells. *J Lipid Res* **38**: 160–172

Berra B, Colombo I, Monteggia E, Montorfano G, Moretti S, Rapelli S and Sottocornola E (1997) Glycosphingolipid expression in solid tumours and transformed cell lines. *Indian J Biochem Biophys* **34**: 170–177

Brigande JV, Platt FM and Seyfried TN (1998) Inhibition of glycosphingolipid biosynthesis does not impair growth or morphogenesis of the postimplantation mouse embryo. *J Neurochem* **70**: 871–882

Chang F, Li R and Ladisch S (1997) Shedding of gangliosides by human medulloblastoma cells. *Exp Cell Res* **234**: 341–346

Cotterchio M and Seyfried TN (1993) The influence of ImuVert, a biological response modifier, on the growth and ganglioside composition of murine neural tumors. *Mol Chem Neuropathol* **20**: 163–172

Cotterchio M and Seyfried TN (1994) Serum gangliosides in mice with metastatic and non-metastatic brain tumors. *J Lipid Res* **35**: 10–14

Cox T, Lachmann R, Hollak C, Aerts J, van Weely S, Hrebicek M, Platt F, Butters T, Dwek R, Moyses C, Gow I, Elstein D and Zimran A (2000) Novel oral treatment of Gaucher's disease with N-butyldeoxynojirimycin (OGT 918) to decrease substrate biosynthesis. *Lancet* **355**: 1481–1485

Davis FG, McCarthy BJ, Freels S, Kupelian V and Bondy ML (1999) The conditional probability of survival of patients with primary malignant brain tumors: surveillance, epidemiology, and end results (SEER) data. *Cancer* **85**: 485–491

Ecsedy JA, Yohe HC, Bergeron AJ and Seyfried TN (1998) Tumor-infiltrating macrophages contribute to the glycosphinglipid composition of brain tumors. *J Lipid Res* **39**: 2218–2227

Ecsedy JA, Holthaus KA, Yohe HC and Seyfried TN (1999) Expression of mouse sialic acid on gangliosides of a human glioma grown as a xenograft in SCID mice. *J Neurochem* **73**: 254–259

El-Abbadi M and Seyfried TN (1994) Influence of growth environmental on the ganglioside composition of an experimental mouse brain tumor. *Mol Chem Neuropathol* **21**: 273–285

Fathallah-Shaykh H (1999) New molecular strategies to cure brain tumors. *Arch Neurol* **56**: 449–453

Fischl MA, Resnick L, Coombs R, Kremer AB, Pottage JC, Jr Fass RJ, Fife KH, Powderly WG, Collier AC, Aspinall RL, Smith SL, Kowalski KG and Wallemark C-B (1994) The safety and efficacy of combination N-butyl-deoxynojirimycin (SC-48334) and zidovudine in patients with HIV-1 infection and 200–500 CD4 cells/mm3. *J Acquir Immune Defic Syndr* **7**: 139–147

Flavin HJ, Wieraszko A and Seyfried TN (1991) Enhanced aspartate release from hippocampal slices of epileptic (El) mice. *J Neurochem* **56**: 1007–1011

Folch J, Lees M and GH Sloane-Stanley (1957) A simple method for the isolation and purification of total lipids from animal tissues. *J Biol Chem* **226**: 497–509

Hakomori S (1996) Tumor malignancy defined by aberrant glycosylation and sphingo (glyco) lipid metabolism. *Cancer Res* **56**: 5309–5318

Harbaugh KS and Black PM (1998) Strategies in the surgical management of malignant gliomas. *Semin-Surg-Oncol* **14**: 26–33

Jeyakumar M, Butters TD, Cortina-Borja M, Hunnam V, Proia RL, Perry VH, Dwek RA and Platt FM (1999) Delayed symptom onset and increased life expectancy in Sandhoff disease mice treated with N-butyldeoxynojirimycin. *Proc Natl Acad Sci USA* **96**: 6388–6393

Karlsson GB, Butters TD, Dwek RA and Platt FM (1993) Effects of the imino sugar N-butyldeoxynojirimycin on the N-glycosylation of recombinant gp120. *J Biol Chem* **268**: 570–576

Kemp RB (1968) Effect of the removal of cell surface sialic acids on cell aggregation in vitro. *Nature* **218**: 1255–1256

Liu YY, Han TY, Giuliano AE and Cabot MC (1999) Expression of glucosylceramide synthase, converting ceramide to glucosylceramide, confers adriamycin resistance in human breast cancer cells. *J Biol Chem* **274**: 1140–1146

Manfredi MG, Lim S, Claffey KP and Seyfried TN (1999) Gangliosides influence angiogenesis in an experimental mouse brain tumor. *Cancer Res* **59**: 5392–5397

Merzak A, Koochekpour S, McCrea S, Roxanis Y and Pilkington GJ (1995) Gangliosides modulate proliferation, migration, and invasiveness of human brain tumor cells in vitro. *Mol Chem Neuropathol* **24**: 121–135

Platt FM and Butters TD (1995) Inhibitors of glycosphingolipid biosynthesis. *Trends Glycosci Glycotech* **7**: 495–511

Platt FM and Butters TD (1998) New therapeutic prospects for the glycosphingolipid lysosomal storage diseases. *Biochem Pharmacol* **56**: 421–430

Platt FM, Neises GR, Dwek RA and Butters TD (1994) N- butyldeoxynojirimycin is a novel inhibitor of glycolipid biosynthesis. *J Biol Chem* **269**: 8362–8365

Platt FM, Neises GR, Reinkensmeier G, Townsend MJ, Perry VH, Proia RL, Winchester B, Dwek RA and Butters TD (1997a) Prevention of lysosomal storage in Tay-Sachs mice treated with N- butyldeoxynojirimycin. *Science* **276**: 428–431

Platt FM, Reinkensmeier G, Dwek RA and Butters TD (1997b). Extensive glycosphingolipid depletion in the liver and lymphoid organs of mice treated with N-butyldeoxynojirimycin. *J Biol Chem* **272**: 19365–19372
Prados MD and Levin V (2000) Biology and treatment of malignant glioma. *Semin Oncol* **27**: 1–10
Radin NS (1999) Chemotherapy by slowing glucosphingolipid synthesis. *Biochem Pharmacol* **57**: 589–595
Ranes MK, Manfredi MG, El-Abbadi MM, Platt FM and Seyfried TN (2000) N-Butyldeoxynojirmycin reduces growth rate and ganglioside content in mouse brain tumors. *Proc Amer Assoc Cancer Res* **41**: 258
Rubin R, Sutton CH and Zimmerman HM (1968) Experimental ependymoblastoma (fine structure). *J Neuropathol Exp Neurol* **27**: 421–438
Sandhoff K and van Echten G (1994) Ganglioside metabolism: enzymology, topology and regulation. *Prog Brain Res* **101**: 17–29
Schmidt CW (1998) Childhood cancer: A growing problem. *Environ Health Perspect* **106**: A18–A23
Seyfried TN (2001) Perspectives on brain tumor formation involving macrophages, glia, and neural stem cells. *Persp Biol Med* in press
Seyfried TN, Glaser GH and Yu RK (1978) Cerebral, cerebellar, and brain stem gangliosides in mice susceptible to audiogenic seizures. *J Neurochem* **31**: 21–27
Seyfried TN, Yu RK, Saito M and Albert M (1987) Ganglioside composition of an experimental mouse brain tumor. *Cancer Res* **47**: 3538–3542
Seyfried TN, El-Abbadi M and Roy ML (1992) Ganglioside distribution in murine neural tumors. *Mol Chem Neuropathol* **17**: 147–167
Seyfried TN, Novikov AM, Irvine RA and Brigande JV (1994). Ganglioside biosynthesis in mouse embroys: sialyltransferase IV and the asialo pathway. *J Lipid Res* **35**: 993–1001
Seyfried TN, El-Abbadi M, Ecsedy JA, Bai HW and Yohe H (1996) Influence of host cell infiltration on the glycolipid content of mouse brain tumors. *J Neurochem* **66**: 2026–2033
Seyfried TN, El-Abbadi M, Ecsedy JA, Griffin ME and Yohe HC (1998) Ganglioside composition of a mouse brain tumor grown in the severe combined immunodeficiency (SCID) mouse. *Mol Chem Neuropathol* **33**: 27–37
Shapiro WR (1999) Current therapy for brain tumors: back to the future. *Arch Neurol* **56**: 429–432
Svennerholm L (1957) Quantitative estimation of sialic acids II. A colorimetric resorcinol-hydrochloric acid method. *Biochim Biophys Acta* **24**: 604–611
Weindruch R and Walford RL (1988) *The retardation of aging and disease by dietary restriction* Thomas: Springfield, IL.
Yu RK and Ledeen RW (1970) Gas–liquid chromatographic assay of lipid-bound sialic acids: measurement of gangliosides in brain of several species. *J Lipid Res* **11**: 506–516
Yung WK, Albright RE, Olson J, Fredericks R, Fink K, Prados MD, Brada M, Spence A, Hohl RJ, Shapiro W, Glantz M, Greenberg H, Selker RG, Vick NA, Rampling R, Friedman H, Phillips P, Bruner J, Yue N, Osoba D, Zaknoen S and Levin VA (2000) A phase II study of temozolomide vs. procarbazine in patients with glioblastoma multiforme at first relapse. *Br J Cancer* **83**: 588–593
Zeng G, Gao L, Birkle S and Yu RK (2000) Suppression of ganglioside GD3 expression in a rat F-11 tumor cell line reduces tumor growth, angiogenesis, and vascular endothelial growth factor production. *Cancer Res* **60**: 6670–6676
Zimmerman HM (1982) Production of brain tumors with aromatic hydrocarbons. *Ann N Y Acad Sci* **381**: 320–324

 *© 2001 Cancer Research Campaign*
